# No Association of Early-Onset Breast or Ovarian Cancer with Early-Onset Cancer in Relatives in *BRCA1* or *BRCA2* Mutation Families

**DOI:** 10.3390/genes12071100

**Published:** 2021-07-20

**Authors:** Marion Imbert-Bouteille, Carole Corsini, Marie-Christine Picot, Lucas Mizrahy, Sandrine Akouete, Helena Huguet, Frédéric Thomas, David Geneviève, Patrice Taourel, Marc Ychou, Virginie Galibert, Chloé Rideau, Karen Baudry, Tatiana Kogut Kubiak, Isabelle Coupier, Rémy Hobeika, Yvette Macary, Alain Toledano, Jérôme Solassol, Antoine Maalouf, Jean-Pierre Daures, Pascal Pujol

**Affiliations:** 1 Cancer Genetics Department, CHU Montpellier, Université Montpellier, 34295 Montpellier, France; m-imbert-bouteille@chu-montpellier.fr (M.I.-B.); c-corsini@chu-montpellier.fr (C.C.); lucasmizrahy@gmail.com (L.M.); v-galibert@chu-montpellier.fr (V.G.); c-rideau@chu-montpellier.fr (C.R.); k-baudry@chu-montpellier.fr (K.B.); t-kogutkubiak@chu-montpellier.fr (T.K.K.); i-coupier@chu-montpellier.fr (I.C.); conseilgenetiqueliban@hotmail.com (R.H.); conseilgenetiquelb@gmail.com (Y.M.); 2 Clinical Research and Epidemiology Unit, CHU Montpellier, Université Montpellier, 34295 Montpellier, France; mc-picot@chu-montpellier.fr (M.-C.P.); m-akouete@chu-montpellier.fr (S.A.); h-huguet@chu-montpellier.fr (H.H.); 3 Medecine Department, School Medecine, Medecine College, Federal University of Juiz de Fora, Juiz de Fora 36036-900, Brazil; 4 CNRS, Laboratory CREEC, MIVEGEC, IRD, 34394 Montpellier, France; frederic.thomas2@ird.fr; 5 Genetics Department, CHU Montpellier, Université Montpellier, 34295 Montpellier, France; d-genevieve@chu-montpellier.fr; 6 Société Française de Médecine Prédictive et Personnalisée, SFMPP, 34295 Montpellier, France; 7 Imagerie médicale, Montpellier, Université Montpellier, 34295 Montpellier, France; p-taourel@chu-montpellier.fr; 8 Department of Oncology, ICM-Val d’Aurelle Cancer Center, 34298 Montpellier, France; Marc.Ychou@icm.unicancer.fr; 9 Genetics Department, French Hospital du Levant, Beirut 50-226, Lebanon; dr.a.maalouf@hotmail.com; 10 Oncology Department, Clinique Hartmann, 92309 Levallois, France; alain.toledano@gmail.com; 11 Department of Pathology and Onco-Biology, IRCM, ICM, CHU Montpellier, INSERM, Université Montpellier, 34295 Montpellier, France; j-solassol@chu-montpellier.fr; 12 Biostatistics Department, University of Montpellier I, CHU Montpellier, 34295 Montpellier, France; jp-daures@chu-montpellier.fr

**Keywords:** breast cancer, ovarian cancer, *BRCA1*, *BRCA2*, early-onset

## Abstract

According to clinical guidelines, the occurrence of very early-onset breast cancer (VEO-BC) (diagnosed ≤ age 30 years) or VEO ovarian cancer (VEO-OC) (diagnosed ≤ age 40 years) in families with *BRCA1* or *BRCA2* mutation (*BRCAm*) prompts advancing the age of risk-reducing strategies in relatives. This study aimed to assess the relation between the occurrence of VEO-BC or VEO-OC in families with *BRCAm* and age at BC or OC diagnosis in relatives. We conducted a retrospective multicenter study of 448 consecutive families with *BRCAm* from 2003 to 2018. Mean age and 5-year–span distribution of age at BC or OC in relatives were compared in families with or without VEO-BC or VEO-OC. Conditional probability calculation and Cochran–Mantel–Haenszel chi-square tests were used to investigate early-onset cancer occurrence in relatives of VEO-BC and VEO-OC cases. Overall, 15% (19/245) of families with *BRCA1m* and 9% (19/203) with *BRCA2m* featured at least one case of VEO-BC; 8% (37/245) and 2% (2/203) featured at least one case of VEO-OC, respectively. The cumulative prevalence of VEO-BC was 5.1% (95% CI 3.6–6.6) and 2.5% (95% CI 1.4–3.6) for families with *BRCA1m* and *BRCA2m*, respectively. The distribution of age and mean age at BC diagnosis in relatives did not differ by occurrence of VEO-BC for families with *BRCA1m* or *BRCA2m*. Conditional probability calculations did not show an increase of early-onset BC in VEO-BC families with *BRCA1m* or *BRCA2m*. Conversely, the probability of VEO-BC was not increased in families with early-onset BC. VEO-BC or VEO-OC occurrence may not be related to young age at BC or OC onset in relatives in families with *BRCAm*. This finding—together with a relatively high VEO-BC risk for women with *BRCAm*—advocates for MRI breast screening from age 25 regardless of family history.

## 1. Introduction

Women who carry the *BRCA1* and *BRCA2* germline pathogenic variant (*BRCAm*) have a high lifetime risk of breast cancer (BC) and ovarian cancer (OC) [1,2,3,4]. Early-onset BC (EO-BC) and EO-OC are well-known key clinical features of hereditary BC and OC syndrome related to *BRCA1* and *BRCA2*. Women with *BRCAm*, especially *BRCA1* mutation carriers, are at increased risk of EO-BC and EO-OC; the cumulative risk of EO-BC (<40 years old) ranges from 12% to 25%, and that of EO-OC (<50 years old) ranges from 8% to 14% [5,6,7,8,9,10,11].

Reliable age-specific cancer risk estimates are key points in deciding when cancer screening and other cancer risk-reducing strategies should start in young women with *BRCAm*. Previously published studies reporting frequency data for very early-onset BC (VEO-BC, defined here as a diagnosis at <30 years old) and VEO-OC (defined here as a diagnosis at <40 years old) vary widely [8,9,10,11]. According to the largest studies, cumulative incidence estimates of VEO-BC before age 30 ranged from 0.65% to 5.9% for women with *BRCA1m* and from 0.7% to 4.8% with *BRCA2* [8,9,10,11]. Cumulative incidence estimates of VEO-OC before age 40 ranged from 1.8% to 2.3% with *BRCA1* and 0.09% to 0.3% with *BRCA2*.

According to international guidelines for management and prevention in women with *BRCAm*, BC screening with breast MRI should start at age 25 (National Comprehensive Cancer Network [NCCN]; European Society for Medical Oncology [ESMO]) or age 30 (National Institute for Health and Care Excellence; National Institute of Cancer [INCa]) and women should consider risk-reducing salpingo-oophorectomy (RRSO) from age 35 to 40 (NCCN; ESMO) or at age 40 (INCa) and upon child-bearing completion [12,13,14,15,16,17]. According to most guidelines, this starting age should be personalized based on family history [12,13,14,15,16,17]. However, which family history clinical features affect the risk of VEO-BC and/or VEO-OC for women with *BRCAm* remain unclear and mostly empirically assessed. Most guidelines also state that with a family history of EOa or VEO-BC, the age for starting breast MRI screening should be personalized and considered earlier, 5 to 10 years, before the youngest age at BC diagnosis in the family. Implementing this guideline assumes that if the family history includes BC diagnosed under age 35, the age to start breast MRI screening should be tailored downward, before 30 or before 25 [12,13,14,15,16,17].

Nevertheless, individualization of the starting age of prevention—based on family history and the youngest age at BC diagnosis—is a widespread empirical approach, mostly based on common sense and psychological factors. However, data supporting a link between EO cancer and the age of other cancers diagnosed in a family are weak [8,10,18,19,20,21,22,23,24,25]. Indeed, family-based factors other than the *BRCAm* variant itself—including the number of relatives with BC or OC and the age at diagnosis of relatives—affect the magnitude of the lifetime risk of BC and OC in women with *BRCAm* [10,18,24,26]. Regardless, the effect of the age at cancer diagnosis of affected relatives as a relevant predictor of a recurrent risk of VEO cancer in a family was not demonstrated [10,18,19,20,21,22,23,24,25].

Some genotype–phenotype correlations have been identified, highlighting some regions of *BRCA1* and *BRCA2* and types of pathogenic variants as features related to increased lifetime risk of BC or OC regions; however, these are not currently used for personalizing screening and preventive strategies [10,27,28,29,30]. Little is known about genotypic *BRCA1* and *BRCA2* features that might be related to earlier onset of cancer.

This study aimed to assess the relation between VEO-BC or VEO-OC in women with *BRCAm* and age at BC and OC diagnosis in relatives. 

## 2. Materials and Methods

### 2.1. Study Population

We studied 450 consecutive families with a *BRCAm* mutation over 15 years in seven centers (Montpellier University Hospital, Montpellier, France; Montpellier Cancer Institute, Montpellier, France; General Hospital of Béziers, Béziers, France; General Hospital of Perpignan, Perpignan, France; Montpellier Breast Institute, Montpellier, France; Hartmann Clinics, Neuilly sur Seine, France; Institut Franco Britannique, Levallois, France). 

Eligible families were identified in a local medical comprehensive database as families, including at least one individual, male or female, with genetic proof of carriage of a *BRCAm* who were referred to one of the seven cancer genetics units mentioned above between 1 January 2003 and 31 January 2019. Families were excluded if the medical record of the individual referred to the Montpellier genetics department was unavailable or did not at least mention first-degree relatives in a medical pedigree (*n* = 2).

For wording convenience, individuals with genetically proven *BRCAm* carriage referred to the Montpellier genetics department are hereafter called cases but could also be a proband (first individual in a family with proof of carriage of the *BRCAm*) or an affected or unaffected relative who underwent a targeted genetic test and had proof of carriage of *BRCAm*.

### 2.2. Data Collection

For each included family, the following data were collected: *BRCAm* status (compliant with the national laboratory standards described in Lesueur et al. [30]); *BRCAm* characteristics (Human Genome Variation Society nomenclature according to sequence references *BRCA1* [NM_007294.3] and *BRCA2* [NM_000059.3]); genetic family anonymous identification number; type and age at cancer onset, including BC and OC and all other types of cancers in the case (as defined above); family make-up with first- to fifth-degree relative affected and unaffected relatives of the case, if available; and cancer data for relatives (type and age at onset for each diagnosis). Available cancer data were ascertained by medical record validation of self-reported cancer diagnoses.

Parents, children and siblings were defined as first-degree relatives; grandparents, grandchildren, uncles, aunts, nephews as second-degree relatives; first cousins, great grandparents, grand aunts, grand uncles, grand nephews as third-degree relatives; second cousins, great grandchildren as fourth-degree relatives; third cousins, great grand aunts and great grand uncles as fifth-degree relatives.

### 2.3. Calculation of Number of BRCAm Carrier Women 

The total number of individuals was calculated by summing the included family make-up. The number of *BRCAm* carrier women among relatives in each family was estimated according to the following formula, accounting for the autosomal dominant pattern of *BRCA1/2* inheritance: *n*_1_/2 + *n*_2_/4 + *n*_3_/8 + *n*_4_/16 + *n*_5_/32, where *n*_1_, *n*_2_, *n*_3_, *n*_4_ and *n*_5_ are the number of first-, second-, third-, fourth- and fifth-degree female relatives, respectively, aged ≥20 years at the latest pedigree update, within the family side with proof of carriage of the pathogenic variant (as previously defined [31]). If the family side carrying the pathogenic variant lacked genetic proof, the number of female relatives within each degree of the family was extracted and a 0.5 coefficient was applied. By summing the estimated number of *BRCAm* carrier female relatives in each family and the total number of female cases, we obtained the estimated total number of *BRCAm* carrier women.

### 2.4. Outcome Measures and Statistical Analysis

#### 2.4.1. Families According to VEO Cancer

Families including at least one woman with BC diagnosed at ≤30 years old were considered VEO-BC families. Conversely, families with no woman diagnosed with BC at ≤30 years old were considered no VEO-BC families. Likewise, families with at least one woman with OC diagnosed at ≤40 years old and those with no VEO-OC women were considered VEO-OC and no VEO-OC families, respectively. Families with no VEO-BC or VEO-OC women were considered no VEO-BC/no VEO-OC families.

#### 2.4.2. Cumulative VEO-BC and VEO-OC Risk

Cumulative VEO-BC and VEO-OC risk was calculated separately for VEO-BC and VEO-OC as the percentage of women with a diagnosis of at least one VEO cancer among the estimated number of *BRCA1m* or *BRCA2m* carrier women.

#### 2.4.3. Mean Age at BC and OC Diagnosis in Relatives

In each family, the mean age at BC diagnosis of affected female relatives was calculated, considering the youngest age at diagnosis for women with multiple BC diagnoses. In no VEO-BC families, age at BC diagnosis of all affected females was considered. In VEO-BC families with only one VEO-BC cases, age at BC diagnosis of all affected females was considered, except for age at diagnosis of the female with the VEO-BC. In VEO-BC families with ≥2 women with VEO-BC diagnosed within the family, age at BC diagnosis of all affected females was considered, except for age at diagnosis of the latest VEO-BC. Families with missing data for age at diagnosis were not included in calculations.

One value for mean age at BC and OC diagnosis in relatives was obtained for each family.

Medians, quartiles and averages of these mean age values were then calculated for VEO BC families, no VEO BC families, VEO OC families and no VEO OC families. 

A two-sided Wilcoxon–Mann–Withney test was used to compare the average of mean age at diagnosis within each family between VEO BC (or OC) families and no VEO BC (or OC) families.

A mixed-effects model was also used to analyze the effect of age at diagnosis of BC (or OC) of relatives of women with VEO-BC (or OC). Each family corresponding to a cluster (including all members of the family) was included as a random effect. The group VEO-BC versus no VEO-BC families was included as a fixed effect. 

#### 2.4.4. Distribution of Age at BC and OC Diagnosis in Relatives

The distribution of age according to early BC diagnosis was calculated in 5-year age groups, separately for two subsets of families, VEO-BC and no VEO-BC, as the ratio of women with a diagnosis of BC in each age group to the total number of *BRCAm* carrier women of the family subset. A comparison of these distributions in four categories of age (age 31 to 35 years, 36 to 40, 41 to 50, >51) involved the chi-square or Fisher exact test. The calculations and comparisons were the same for VEO-OC, with the following four categories of age: 41 to 45 years, 46 to 50, 51 to 60, >61.

#### 2.4.5. Relation between VEO-BC Occurrence and EO-BC in Relatives 

According to several guidelines, if the family history includes a case of EO-BC, breast MRI screening for relatives of *BRCAm* carrier women should start 5 to 10 years before the youngest age of BC diagnosis in the family [12,13,14,15,16,17]. This recommendation assumes that family history of VEO-BC is a potential predictor of increased risk of EO-BC within the family and the reverse. Therefore, we hypothesized that, within families, VEO-BC (occurring before age 30) could be related to EO-BC in relatives (occurring before age 35 years) and the reverse.

We thus calculated the probability of VEO-BC occurring under the following conditions: no BC occurred in the 31–35 age class in the family; at least one BC case occurred in the 31–35 age class in the family; and one BC case, two BC cases and three BC cases in the 31–35 age class occurred in the 31–35 age class in the family.

Conversely, we calculated the probability of a BC case occurring in the 31–35 age class in the family under the following conditions: no VEO-BC case occurred in the family, at least one VEO-BC case occurred, and one VEO-BC case and two VEO-BC cases occurred.

All probabilities were estimated using multiple conditional probabilities [P(A/B∩C) = P((A/B)/C)]. 

The analysis of the relation between the presence of women with BC diagnosed before age 30 years (VEO-BC) and the percentage of women with BC diagnosed between age 31 to 35 (EO-BC) was stratified by total number of BC cases in the family with a Cochran–Mantel–Haenszel chi-square test. All measures, calculations and analyses involved *BRCA1* and *BRCA2* separately (and for *BRCA1/2* together, if mentioned).

All statistical analyses were performed with SAS 9.2.2 (SAS Institute, Cary, NC, USA). All *p*-values were based on two-sided tests and were considered statistically significant at *p* ≤ 0.05.

## 3. Results

### 3.1. Study Population

A total of 450 families were eligible and 448 families were included (2 families excluded because of a lack of available medical data), totalling 11,016 individuals (cases and relatives), 1236 BC and 280 OC cases and 1614 women carrying a *BRCAm* (genetically proven female cases and estimated number of female relative *BRCA1m* carriers). Overall, 245 families featured *BRCA1m* and 203 featured *BRCA2m*.

The mean (SD) number of individuals in families was 24.5 (12.04) (median 22.0 [Q1–Q3 17–30]; range 3–81). The mean number of *BRCAm* carrier women in a family was 3.6 (1.4) (median 3.3 [Q1–Q3 2.6–4.3]; range 0.5–12). Family and patient characteristics are in Supplementary Table S1.

### 3.2. VEO-BC and VEO-OC Families

**Figure 1:**
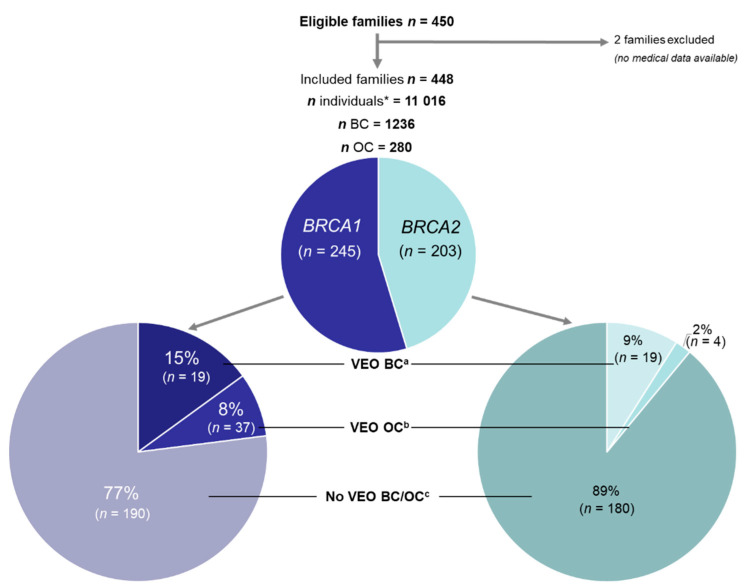
Flowchart of study population and population subsets according to very early-onset breast cancer (VEO-BC) or VEO ovarian cancer (VEO-OC) cases. (*n*): number of families in each population subset. * Number of all men and women, affected and unaffected, individuals in included families. When the *BRCAm* inheritance family side was known, only individuals from this inheritance family side were counted. ^a^ Families with at least one woman with VEO-BC (age at diagnosis <30 years). ^b^ Families with at least one woman with VEO-OC (age at diagnosis <40 years). ^c^ Families with no women with a diagnosis of VEO-BC or VEO-OC.

In total, 78 of the 448 (17.4%) families had at least one VEO cancer case (BC and/or OC): 15% of *BRCA1* families included a VEO-BC case and 8% a VEO-OC case, with 9% and 2%, respectively, for *BRCA2* families (Figure 1).

In *BRCA1* families, VEO-BC families mostly included only one case of VEO-BC (30 families; 12%). Seven families (3%) included two cases of VEO-BC (Supplementary Table S1). Age at VEO-BC diagnosis ranged from 21 to 30 years; 38 of 44 (86%) women with VEO-BC had a diagnosis between age 26 and 30 years. 

In *BRCA2* families, all VEO-BC families included only one case of VEO-BC. Age at VEO-BC diagnosis ranged from 23 to 30 years: 17 of 19 (89%) women with VEO-BC had a diagnosis between age 26 and 30 years.

Amongst *BRCA1m* families, 2 of 19 families included 2 women with VEO-OC and one family included 3 women with VEO-OC. Age at VEO-OC diagnosis ranged from 21 to 40 years: 13 of 23 (57%) women had a diagnosis between age 36 and 40 years, 26% between 31 and 35 years and 17% <30 years. In *BRCA2* families, all 4 VEO-OC families included only one case each of VEO-OC, with one VEO-OC case diagnosed between age 36 and 40 years, 2 between 31 and 35 years and one <30 years.

### 3.3. Cumulative Risk of VEO-BC and VEO-OC 

The cumulative risk of VEO-BC was 5.1% (95% CI 3.6–6.6) in *BRCA1m* carrier women and 2.5% (95% CI 1.4–3.6) in *BRCA2m* carrier women. The corresponding numbers for VEO-OC were 2.7% (95% CI 1.6–3.8) and 0.5% (95% CI 0.0–1.0) (Table 1).

### 3.4. Age at BC and OC Diagnosis in Relatives of BRCAm Carrier Women with VEO-BC or VEO-OC

#### 3.4.1. Mean Age at BC and OC Diagnosis in Relatives

**Figure 2:**
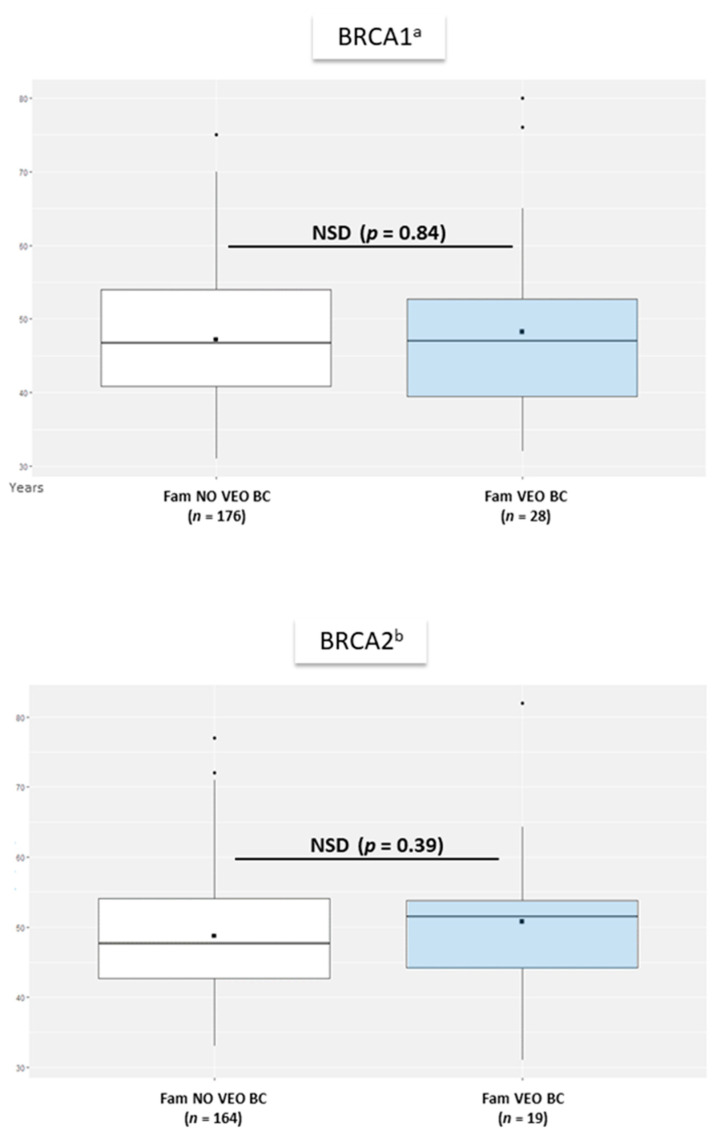
Average of mean age at BC diagnosis in female relatives of women with VEO-BC versus no VEO-BC. ^a^: among *BRCA1* mutation (*BRCA1m*) carrier women. ^b^: among *BRCA2m* carrier women. Fam. VEO-BC: families with at least one VEO-BC. Fam. NO VEO-BC: Families without any VEO-BC. (*n*): number of families with available data for age at first BC diagnosis in women. NSD: nonsignificant difference (two-sided Wilcoxon–Mann–Withney test). Horizontal line is median, box edges are 1st and 3rd quartile and whiskers are range of age at diagnosis of BC in relatives in each family. Central black square is the mean average age at diagnosis of BC in relatives in each family.

Average of mean age at BC diagnosis of female relatives of a *BRCAm* carrier with VEO-BC did not statistically differ from average of mean age at BC diagnosis of affected women of no VEO-BC *BRCAm* families, in both *BRCA1m* and *BRCA2m* families (Figure 2).

**Figure 3:**
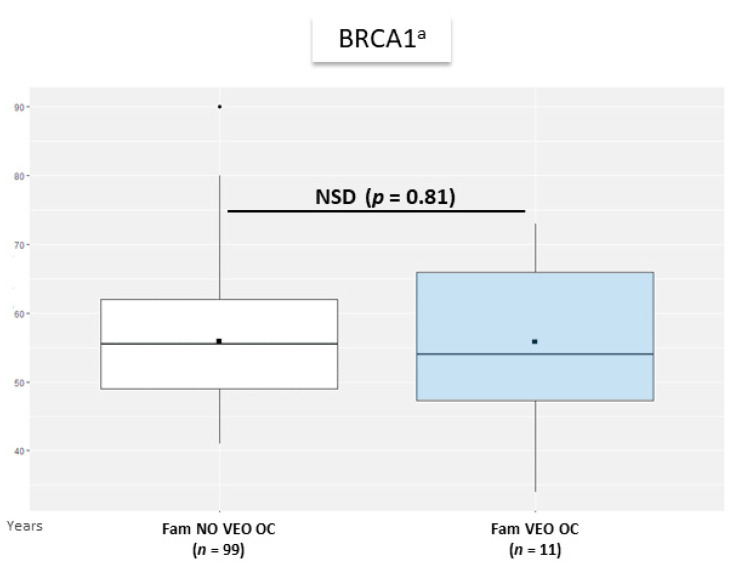
Average of mean age at OC diagnosis of female relatives of women with VEO-OC versus no VEO-OC among *BRCA1m* carrier women. Fam. VEO-OC: families with at least one or more VEO-OC case. Fam. NO VEO-OC: families without VEO-OC cases. (*n*): number of families with available data for age at first OC diagnosis in women. ^a^: among *BRCA1m* carrier women. Horizontal line is median, box edges are 1st and 3rd quartile and whiskers are range of age at diagnosis of BC in relatives in each family. Central black square is the mean average age at diagnosis of BC in relatives in each family.

For VEO-OC, average of mean age at OC diagnosis did not significantly differ between female relatives of women with VEO-OC and no VEO-OC (Figure 3).

Mean age at cancer diagnosis in each family was 47.0 and 54.0 years for BC and OC, respectively, in female relatives of VEO cancer *BRCA1* families, and was 46.8 and 55.5 years, respectively for BC and OC in no VEO cancer *BRCA1* families. Mean age at cancer diagnosis in each family was 51.5 and 56.0 years for BC and OC, respectively, in female relatives of VEO cancer *BRCA2* families, and was 47.7 and 61.0 years, respectively, for BC and OC in no VEO cancer *BRCA2* families (not represented, insufficient data for Wilcoxon–Mann–Withney test, details shown in Supplementary Table S2). Among the 4 *BRCA2* families with a VEO-OC case, one family had another diagnosis of OC (age at diagnosis 56 years).

#### 3.4.2. Mixed-Effects Model

For *BRCA1* families, mean age at BC diagnosis of female relatives in each family was older in VEO-BC than no VEO-BC families (0.1 year older), with no significant effect of VEO-BC occurrence on age at BC diagnosis of relatives (*p* = 0.93) in the mixed-effect model. Mean age at OC diagnosis of female relatives in each family was younger in VEO-OC than no VEO-OC families (2.6 years older), with no significant effect of VEO-OC occurrence on age at OC diagnosis of relatives (*p* = 0.36).

For *BRCA2*, mean age at BC diagnosis of female relatives in each family was older in VEO-BC than no VEO-BC families (3.6 years older), with a significant fixed effect of VEO-BC occurrence in the family on the age at BC diagnosis of relatives (*p* = 0.02). (Details shown in Supplementary Table S2).

The mixed model could not be applied for age at OC diagnosis of relatives in *BRCA2* families because of insufficient number of VEO-OC families (one *BRCA2* family with VEO-OC and no relative with OC diagnosed in this family).

#### 3.4.3. Distribution of Ages at BC and OC Diagnosis in Relatives 

**Figure 4:**
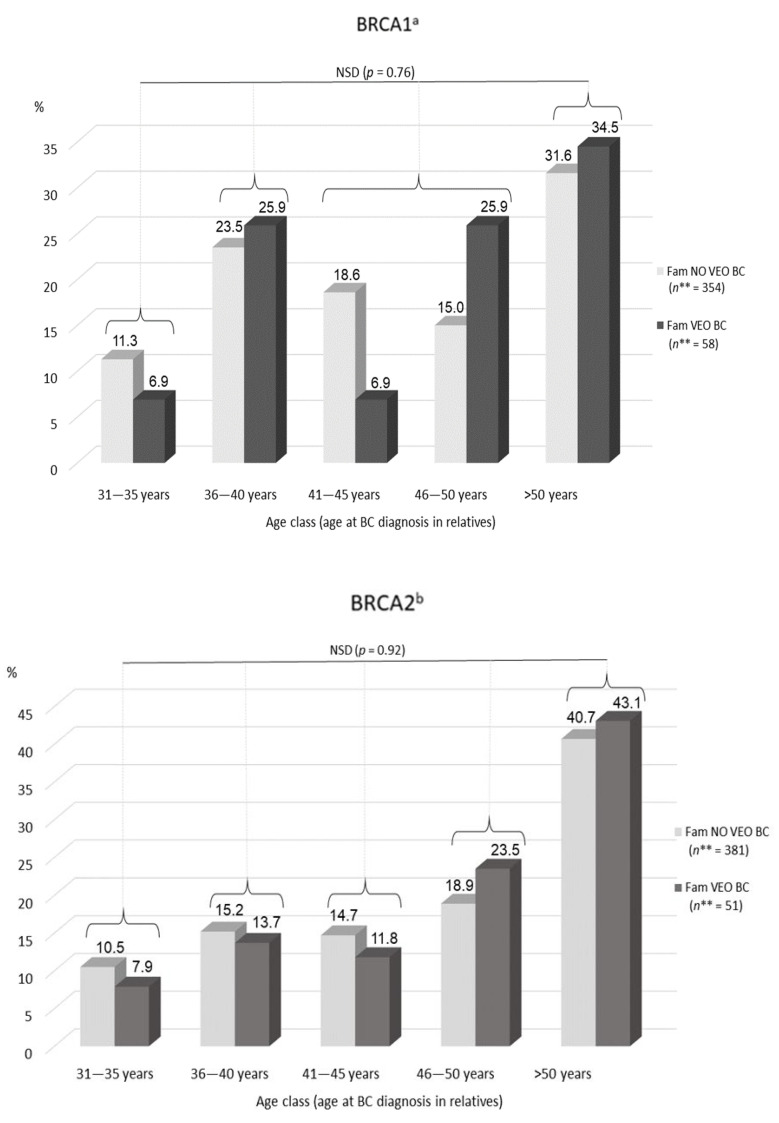

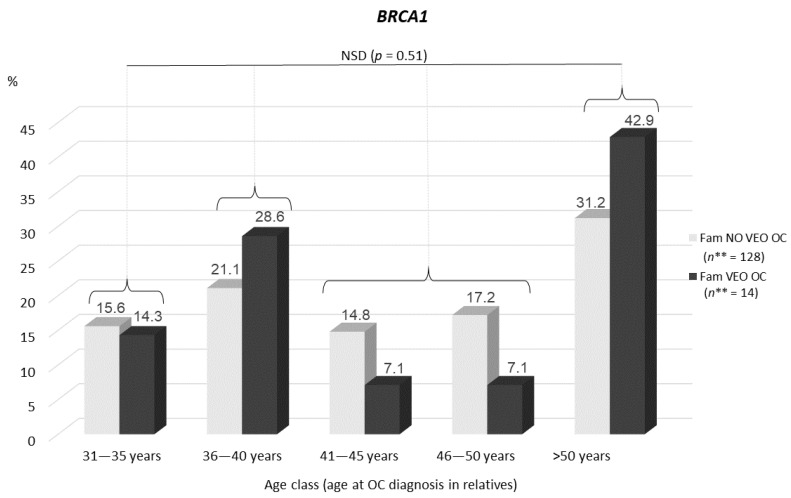
Distribution of ages at BC and OC diagnosis of relatives; comparison of families with VEO cancer and no VEO cancer. Fam. VEO-BC: families including at least one VEO-BC case; Fam. VEO-OC: families with at least one VEO-OC case; Fam. NO VEO-BC: families with no VEO-BC case; Fam. NO VEO-OC: families with no VEO-OC case. n**: Number of women with available data for age at BC diagnosis. Of note: for women with multiple BC diagnoses, the youngest age at diagnosis was considered. ^a^: among *BRCA1m* carrier women. ^b^: among *BRCA2m* carrier women. NSD: non-significant difference.

In *BRCA1* families, the distribution of ages at BC diagnosis of female relatives did not differ between families with and without VEO-BC (*p* = 0.76) (Figure 4). In *BRCA2* families, neither distribution differed (*p* = 0.92). 

In *BRCA1* families, the distribution of ages at OC diagnosis of female relatives did not differ between families with and without VEO-OC (*p* = 0.51). In *BRCA2* families, the distribution and comparisons were not analyzed because of an insufficient number of VEO-OC families (*n* = 1) (details shown in Supplementary Table S3).

### 3.5. Relation between VEO-BC Occurrence and Early-Onset BC (EO-BC) in Relatives 

#### 3.5.1. Conditional Probability of VEO-BC by Occurrence of EO-BC 

In *BRCA1* families, the probability of VEO-BC in families with at least one EO-BC case diagnosed between age 31 and 35 and and no EO-BC case was 1.34% and 2.32% (difference *p* = 0.22). Conversely, the probability of EO-BC in families with at least one VEO-BC case diagnosed between age 31 and 35 and no VEO-BC case was 1.09% and 2.0% (difference *p* = 0.19).

**Figure 5:**
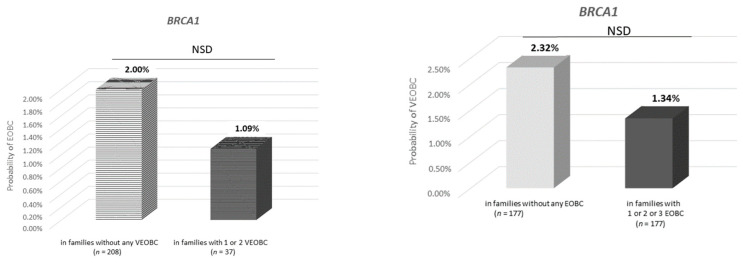

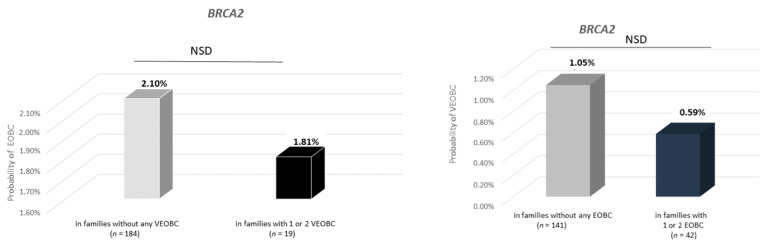
Probability of VEO-BC by number of relatives in the family with BC diagnosed between age 31 and 35. VEO-BC: diagnosed ≤30 years old. EO-BC: early-onset BC (diagnosed between age 31 and 35 years). *n* = number of families. NSD: non-significant difference.

In *BRCA2* families, the probability of VEO-BC in families with at least one EO-BC case diagnosed between age 31 and 35 and no EO-BC case was between 0.59% and 1.05% (difference *p* = 0.32). Conversely, the probability of EO-BC in families with at least one VEO-BC case diagnosed between age 31 and 35 and no VEO-BC case was between 1.09% and 2.0% (difference *p* = 0.76). (Figure 5 and Supplementary Table S4).

#### 3.5.2. Association between EO Cancer Occurrence and Number of VEO Cancer Cases in the Family (Cochran–Mantel–Haenszel Model)

In *BRCA1* families, we found no significant relation between the number of women with EO-BC diagnosed between age 31 and 35 and the number with VEO-BC in the family (*p* = 0.87)—or between the number of women with EO-OC diagnosed between age 41 and 45 and the number of women with VEO-OC in the family (*p* = 0.45).

In *BRCA2* families, we found no significant relation between the number of women with EO-BC diagnosed between age 31 and 35 and the number with VEO-BC in the family (*p* = 0.87) (Supplementary Table S5).

## 4. Discussion

In this multicenter retrospective study involving 450 consecutive families with *BRCAm*, 17% of all families were affected by VEO-BC and/or VEO-OC. In total, 15% and 8% of *BRCA1m* families and 9% and 2% of *BRCA2m* families exhibited VEO-BC and VEO-OC, respectively. VEO-BC and VEO-OC cases in women with *BRCAm* are considered scarce events, but our results support that the magnitude of the occurrence in clinical cancer genetics practice has been underestimated [1,2,5,6,7,8,9,12,15,28].

For women with *BRCA1m* or *BRCA2m*, we estimated a cumulative risk of VEO-BC of nearly 5% and 2.5%, respectively. This finding was consistent with the uppermost part of the range of previous estimates, especially the cumulative incidence recently estimated by Kuchenbaecker et al. in the largest prospective study published to date [8,10,11]: 5.9% (95% CI 3.4–10.1) for BC between age 21 and 30 for *BRCA1* and 4.8% (95% CI 2.0–11.5) for *BRCA2*. 

For VEO-OC, our estimates of cumulative risk also agreed with the estimated incidences in the literature for *BRCA1* and *BRCA2*, and confirmed a meaningful occurrence of such events—but only in women with *BRCA1m* [10].

Our results also highlighted an earliness differential between *BRCA1* and *BRCA2* families. This finding was expected, given the differential pattern of age-specific penetrance of the 2 genes, with *BRCA1m* leading to more frequent BC occurrence under age 40 and OC occurrence under age 50 as compared with *BRCA2m* [8,9,10,11]. We confirmed a higher earliness frequency of cancer onset with *BRCA1* than *BRCA2* for the youngest age groups as well.

Our results did not provide any clue as to a relation between age at diagnosis of relatives of women with VEO cancer and the occurrence of VEO cancer within the family. When considering the age at BC diagnosis in affected relatives of a women with VEO-BC, the mean age at BC diagnosis was similar in families with and without VEO-BC. Nor did we find a difference in age at OC diagnosis in affected relatives in families with and without VEO-OC. Furthermore, the distribution of ages at BC and OC diagnosis of relatives did not differ between families with and without VEO cancer. In the frontier age classes, from age 31 to 40 years, when a difference could have been expected, the proportion of VEO-BC and no VEO-BC families was similar and did not significantly differ. Most of the VEO-OC cases seemed isolated within each family. 

In addition, when we focused on the 31- to 35-year frontier age class at BC diagnosis of affected relatives, neither the absence or presence of BC diagnosis between age 31 and 35 nor the number of such EO-BC diagnoses in a family were significantly related to VEO-BC. Therefore, our data do not support the intuitive concept of starting breast screening 5 to 10 years before the youngest age at BC diagnosis in the family, despite being recommended in several guidelines and commonly applied in clinical routine practice [12,13,14,15,16,17]. Hence, we suggest that family history, especially young age at diagnosis in relatives, might not be a relevant criterion to personalize the starting age of breast screening in women with *BRCAm*.

The magnitude of lifetime risk of BC and OC in women with *BRCAm* has been related to the location of the pathogenic variants in Breast Cancer Cluster Regions and Ovarian Cancer Cluster Regions [10,27,28,29,30,32]. It has also been suggested that some types of variants could be associated with a small but significant effect on age at onset of cancer. In women with *BRCA1m*, nonsense-mediated mRNA decay variants were found to be associated with a 2-year older mean age at BC onset; nonsense variants located in exon 11 were associated with a 2-year earlier mean age at BC and OC onset; and in women with *BRCA2m*, nonsense variants located in exon 11 were associated with reduced lifetime BC risk but a 2.5-year earlier mean age at onset [29]. However, as far as we know, no genotype data allow for a reliable prediction of increased risk of EO or VEO-BC and VEO-OC in women with *BRCAm* [33].

Altogether, our results and the revisited risk of VEO-BC published in the most recent studies might prompt considering starting breast MRI screening from age 25 for all women with *BRCAm*, given that BC onset under age 30, especially between age 25 and 30, is not rare in this population and that no family-based reliable criterion allows for accurately identifying the women who need tailored preventive measures. Moreover, this very young population more frequently has a diagnosis of triple-negative or basal-like tumors than older women [11,34,35,36,37]. These tumor phenotypes benefit from MRI screening [38,39]. The potential aggressiveness of these tumor types also strengthens the benefit that might be expected from such cautious screening [40,41,42,43]. 

Periodic breast MRI, as the most sensitive screening (sensitivity range: 85–93%) for early-stage BC, not exposing the mammary glands to X-rays, is recommended and performed from age 25 in many countries, including the United States, the Netherlands and Poland [13,14,44,45]. Despite a reported ~20% rate of false-positive results at the first breast MRI screening, the actual uptake of breast MRI screening in women with *BRCAm* in these countries is high (>80%), which indicates good acceptability of this preventive program by patients [46,47,48,49]. The cost-effectiveness assessment of screening by breast MRI in women with *BRCAm* was reported as positive, including when starting yearly breast MRI screening from age 25 [50,51,52].

The cumulative risk of VEO-OC we found in women with *BRCA1m* (reaching ~2.5%) addresses a more delicate issue: the optimal age for RRSO, aiming at a compromise between OC risk and preventive surgery-related mortality decrease, child-bearing completion, and the consequences of iatrogenic premature ovarian insufficiency [53,54,55,56,57]. US guidelines state that RRSO should be performed between age 35 to 40 in women with *BRCA1m*, whereas some other guidelines (e.g., the latest national French guidelines or British guidelines) advise it to be performed at age 40, after child-bearing completion [12,13,15,58]. Taking into account an occurrence of VEO-OC in women with *BRCA1m* being not as rare as historically reported, the age of 40 years should not be considered a minimum age to perform RRSO after child-bearing completion when counselling unaffected women with *BRCA1m*. Consequently, our results enhance the need to provide young unaffected women with *BRCAm* with appropriate genetic counselling about parenthood plan completion [59,60]. Fertility preservation could be a relevant additional option [61,62,63,64].

Because our data lead to tempering the reliability of basing individualization of the starting age of preventive measures on family history, they also emphasize the need for refining the understanding of phenotype variability of *BRCA1/2* cancer-susceptibility hereditary syndrome. Genetic and nongenetic modifier factors have been widely explored and provided evidence for impact of environmental factors (mainly lifestyle and estrogen exposure-related factors), a large set of single nucleotide polymorphisms and genotypic features of *BRCA1/2* variants themselves [29,30,64,65,66,67,68,69,70,71,72,73,74,75,76,77,78]. To our knowledge, no *BRCA1/2* genotype–phenotype association with underlying age at cancer onset variability has been clearly identified [65,66,67,68,69,70,71,72,73,74]. One hypothesis for this could be that extreme earliness in cancer onset in women with *BRCAm* might be affected to a greater extent by extrinsic modifiers than pathogenic variant location or type.

Although we conducted a single-country study, which could imply insufficient representativeness, we used comprehensive and consecutive data collection, covering a 15-year time frame, which allowed for a long follow-up of these families in 7 different French cancer genetic centers. This design allowed us to build a dataset including more than 11,000 individuals from 448 *BRCA1/2* families, which supports a good representativeness of women with *BRCAm* in the French population.

The retrospective design is certainly a limitation of this study, because ascertainment bias and biases due to inaccuracies in the reporting of family history could not fully be avoided—despite rigorous checking of data by medical record validation, strengthened by a long-term follow-up of most families.

In this study, the case selection pooled affected probands referred for genetic testing because of personal or family history of cancer according to international standards for *BRCA1/2* testing and unaffected carrier relatives of probands referred for presymptomatic targeted testing. This design could imply overestimation of EO and VEO cancers. However, because of the comprehensiveness of the inclusion of families, we believe that this population represents that in most cancer genetics centers. Moreover, clinical standards for *BRCA1/2* testing have been evolving over the last 15 years, from more stringent criteria toward enlarged criteria [13,79]. This criteria loosening might dilute families with the most aggressive phenotype expression such as VEO cancer in more families with less severe expression or penetrance of the *BRCA1/2*-related cancer susceptibility syndrome. Regardless, our estimates of cumulative risk of VEO-BC and VEO-OC in women with *BRCAm* over 2003 to 2015 and 2016 to 2018 are homogeneous and consistent with the uppermost frequency estimates published so far [10]. In further studies in the general population or in women undergoing a somatic *BRCA1/2* test (e.g., for theranostic purposes related to PARP-inhibitor use) regardless of currently used genetic criteria, frequency data for VEO-BC and VEO-OC could be extracted, which would put our results into perspective [80].

*BRCA1/2* status was not available for all female relatives in our study population (including, for example, deceased relatives). Thus, the number of *BRCAm* carrier women in this study pooled pathogenic variant carriers and estimates. Hence, some of the BC and OC diagnoses of relatives might be phenocopies (i.e., developed in noncarrier women). However, because of the extremely low cumulative incidence of BC under age 30 years (<0.1%) and OC under age 40 years (<0.01%) in the population, phenocopying of VEO cancers is unlikely, and so may not have significantly affected our results [81,82]. Nevertheless, it might have influenced, to a limited extent, the distribution of ages at diagnosis, which we observed in relatives, in particular for the oldest age groups. This distribution might have also been biased because of the inclusion of women who underwent risk-reducing breast and/or ovarian surgery. Although further study could analyze data with this parameter stratified or censured, the risk-reducing mastectomy rate in the French *BRCAm* population is quite low (estimated at 10% to 25%) [12,47,48,49]. Bilateral mastectomy and RRSO are not recommended under age 30 and 40, respectively, according to the national guidelines of the French National Cancer Institute [12]. Hence, the outcome we reported herein (cumulative risk of VEO-BC and VEO-OC) and the distribution of ages in the youngest age groups are unlikely to have been affected in our study.

The follow-up duration and required features for appropriate censoring were not available for all families in this study. Thus, we could not calculate incidence. Nevertheless, the cumulative risk and cumulative incidence are expected to be appropriate epidemiologic indexes, allowing for a meaningful comparison, provided they are used for descriptive outcome purposes, as in this study [83,84].

## 5. Conclusions

This study found no relation between the occurrence of VEO cancers in families with *BRCAm* and age at BC or OC diagnoses of relatives. Thus, these results do not support that EO-BC or EO-OC predicts young age at diagnosis of the corresponding cancer in *BRCAm* carrier relatives and do not advocate for tailoring BC or OC risk-reduction strategies on the basis of EO cancer occurrence in the family. Considering this observation—and because the VEO-BC risk was 5% and 2.5% in women with *BRCA1m* or *BRCA2m*—our study advocates offering breast MRI screening from age 25 to all women with *BRCAm*, regardless of family history.

## Figures and Tables

**Table 1:** Cumulative risk of very early-onset breast cancer (VEO-BC) and VEO ovarian cancer (VEO-OC) in *BRCAm* carrier women.

	Cumulative Prevalence (95% CI)	Comparative Data from Literature: Range of Previously Published Cumulative Incidence of VEO Cancer
VEO BC		
*BRCA1* ^a^	5.1% (3.6–6.6)	0.7–8.7% *
*BRCA2* ^b^	2.5% (2.4–3.6)	0.0–4.8% *
VEO OC		
*BRCA1* ^a^	2.7% (1.6–3.8)	1.1–2.3% *
*BRCA2* ^b^	0.5% (0.01–1.0)	0.1–1.7% *

^a^: among *BRCA1m* carrier women. ^b^: among *BRCA2m* carrier women. *: all ranges mentioned are based on following references: Antoniou et al. [8] (*Am. J. Hum. Genet.*, 2003), Kuchenbaecker et al. [10] (*JAMA*, 2017), Mavaddat et al. [11] (*J. Natl. Cancer Inst.*, 2013).

## Data Availability

The data are available on request from the corresponding author. The data are not publicly available due to University Hospital network safety requirements.

## Supplementary Materials

The following are available online at https://www.mdpi.com/article/10.3390/genes12071100/s1: Table S1. Patient and family characteristics; Table S2. Median age of BC diagnosis in relatives of women with VEO-BC; Table S3. Distribution of age at diagnosis of BC and OC of relatives; Table S4. Conditional probability of VEO-BC and EO-BC 31–35; Table S5. Cochran-Mantel-Haenszel.

## Abbreviations

Women with *BRCAm*	women carrying a germline *BRCA1* or *BRCA2* pathogenic variant
BC	breast cancer
CI	confidence interval
EO	early-onset
ESMO	European Society for Medical Oncology
HR	hazard ratio
INCa	National Institute of Cancer (France)
NCCN	National Comprehensive Cancer Network (USA)
NICE	National Institute for Health and Care Excellence (UK)
OC	ovarian cancer
RR	relative risk
RRSO	risk-reducing salpingo-oophorectomy
VEO	very early-onset

## References

[1] Ford D., Easton D.F., Bishop D.T., Narod S.A., Goldgar D.E. (1994). Risks of cancer in *BRCA1*-mutation carriers. Breast Cancer Linkage Consortium. Lancet.

[2] Ford D., Easton D.F., Stratton M., Narod S., Goldgar D., Devilee P., Bishop D.T., Weber B., Lenoir G., Chang-Claude J. (1998). Genetic heterogeneity and penetrance analysis of the *BRCA1* and *BRCA2* genes in breast cancer families. The Breast Cancer Linkage Consortium. Am. J. Hum. Genet..

[3] Tavtigian S.V., Simard J., Rommens J., Couch F., Shattuck-Eidens D., Neuhausen S., Merajver S., Thorlacius S., Offit K., Stoppa-Lyonnet D. (1996). The complete *BRCA2* gene and mutations in chromosome 13q-linked kindreds. Nat. Genet..

[4] Wooster R., Bignell G., Lancaster J., Swift S., Seal S., Mangion J., Collins N., Gregory S., Gumbs C., Micklem G. (1995). Identification of the breast cancer susceptibility gene *BRCA2*. Nature.

[5] Eccles D., Marlow A., Royle G., Collins A., Morton N.E. (1994). Genetic epidemiology of early onset breast cancer. J. Med. Genet..

[6] Stratton J.F., Thompson D., Bobrow L., Dalal N., Gore M., Bishop D.T., Scott I., Evans G., Daly P., Easton D.F. (1999). The genetic epidemiology of early-onset epithelial ovarian cancer: A population-based study. Am. J. Hum. Genet..

[7] Easton D.F., Ford D., Bishop D.T. (1995). Breast and ovarian cancer incidence in *BRCA1*-mutation carriers. Breast Cancer Linkage Consortium. Am. J. Hum. Genet..

[8] Antoniou A., Pharoah P.D.P., Narod S., Risch H.A., Eyfjord J.E., Hopper J.L., Loman N., Olsson H., Johannsson O., Borg A. (2003). Average risks of breast and ovarian cancer associated with *BRCA1* or *BRCA2* mutations detected in case Series unselected for family history: A combined analysis of 22 studies. Am. J. Hum. Genet..

[9] Chen S., Parmigiani G. (2007). Meta-analysis of *BRCA1* and *BRCA2* penetrance. J. Clin. Oncol..

[10] Kuchenbaecker K.B., Hopper J.L., Barnes D.R., Phillips K.-A., Mooij T.M., Roos-Blom M.-J., Jervis S., van Leeuwen F.E., Milne R.L., Andrieu N. (2017). Risks of Breast, Ovarian, and Contralateral Breast Cancer for *BRCA1* and *BRCA2* Mutation Carriers. JAMA.

[11] Mavaddat N., Peock S., Frost D., Ellis S., Platte R., Fineberg E., Evans D.G., Izatt L., Eeles R.A., Adlard J. (2013). Cancer risks for *BRCA1* and *BRCA2* mutation carriers: Results from prospective analysis of EMBRACE. J. Natl. Cancer Inst..

[12] Doutriaux-Dumoulin I. (2018). Suivi des patientes porteuses d’une mutation des gènes *BRCA1* et 2: Recommandations de l’InCa 2017. Imagerie de la Femme.

[13] Daly M.B., Pilarski R., Axilbund J.E., Buys S.S., Crawford B., Friedman S., Garber J.E., Horton C., Kaklamani V., Klein C. (2014). Genetic/familial high-risk assessment: Breast and ovarian, version 1.2014. J. Natl. Compr. Cancer Netw..

[14] Paluch-Shimon S., Cardoso F., Sessa C., Balmana J., Cardoso M.J., Gilbert F., Senkus E., ESMO Guidelines Committee (2016). Prevention and screening in *BRCA* mutation carriers and other breast/ovarian hereditary cancer syndromes: ESMO Clinical Practice Guidelines for cancer prevention and screening. Ann. Oncol..

[15] (2019). Tools and Resources. Familial Breast Cancer: Classification, Care and Managing Breast Cancer and Related Risks in People with a Family History of Breast Cancer.

[16] Corso G., Magnoni F. (2021). Hereditary Breast Cancer: Translation into Clinical Practice of Recent American Society of Clinical Oncology, American Society of Radiation Oncology, and Society of Surgical Oncology Recommendations. Eur. J. Cancer Prev..

[17] Tung N.M., Boughey J.C., Pierce L.J., Robson M.E., Bedrosian I., Dietz J.R., Dragun A., Gelpi J.B., Hofstatter E.W., Isaacs C.J. (2020). Management of Hereditary Breast Cancer: American Society of Clinical Oncology, American Society for Radiation Oncology, and Society of Surgical Oncology Guideline. J. Clin. Oncol..

[18] Metcalfe K., Lubinski J., Lynch H.T., Ghadirian P., Foulkes W.D., Kim-Sing C., Neuhausen S., Tung N., Rosen B., Gronwald J. (2010). Family History of Cancer and Cancer Risks in Women with *BRCA1* or *BRCA2* Mutations. J. Natl. Cancer Inst..

[19] Semple J., Metcalfe K.A., Lubinski J., Huzarski T., Gronwald J., Armel S., Lynch H.T., Karlan B., Foulkes W., Singer C.F. (2015). Does the age of breast cancer diagnosis in first-degree relatives impact on the risk of breast cancer in *BRCA1* and *BRCA2* mutation carriers?. Breast Cancer Res. Treat..

[20] Tilanus-Linthorst M.M.A., Lingsma H.F., Gareth Evans D., Thompson D., Kaas R., Manders P., van Asperen C.J., Adank M., Hooning M.J., Kwan Lim G.E. (2013). Optimal age to start preventive measures in women with *BRCA1/2* mutations or high familial breast cancer risk. Int. J. Cancer.

[21] Panchal S., Bordeleau L., Poll A., Llacuachaqui M., Shachar O., Ainsworth P., Armel S., Eisen A., Sun P., Narod S.A. (2010). Does family history predict the age at onset of new breast cancers in *BRCA1* and *BRCA2* mutation-positive families?. Clin. Genet..

[22] Cooper J.M. (2010). Factors Associated with Early Versus Late Development of Breast and Ovarian Cancer in BRCA1 and BRCA2 Positive Women.

[23] Soegaard M., Frederiksen K., Jensen A., Høgdall E., Høgdall C., Blaakaer J., Ramus S.J., Gayther S.A., Kjaer S.K. (2009). Risk of ovarian cancer in women with first-degree relatives with cancer. Acta Obstet. Gynecol. Scand..

[24] Stratton J.F., Pharoah P., Smith S.K., Easton D., Ponder B.A. (1998). A systematic review and meta-analysis of family history and risk of ovarian cancer. Br. J. Obstet. Gynaecol..

[25] Pharoah P.D., Day N.E., Duffy S., Easton D.F., Ponder B.A. (1997). Family history and the risk of breast cancer: A systematic review and meta-analysis. Int. J. Cancer.

[26] Begg C.B., Haile R.W., Borg Å., Malone K.E., Concannon P., Thomas D.C., Langholz B., Bernstein L., Olsen J.H., Lynch C.F. (2008). Variation of Breast Cancer Risk Among *BRCA1/2* Carriers. JAMA.

[27] Thompson D., Easton D., Breast Cancer Linkage Consortium (2002). Variation in *BRCA1* cancer risks by mutation position. Cancer Epidemiol. Biomark. Prev..

[28] Thompson D., Easton D.F. (2003). Cancer Incidence in *BRCA1* Mutation Carriers. Obstet. Gynecol. Surv..

[29] Rebbeck T.R., Mitra N., Wan F., Sinilnikova O.M., Healey S., McGuffog L., Mazoyer S., Chenevix-Trench G., Easton D.F., Antoniou A.C. (2015). Association of type and location of *BRCA1* and *BRCA2* mutations with risk of breast and ovarian cancer. JAMA.

[30] Lesueur F., Mebirouk N., Jiao Y., Barjhoux L., Belotti M., Laurent M., Léone M., Houdayer C., Paillerets B.B., Vaur D. (2018). GEMO, a National Resource to Study Genetic Modifiers of Breast and Ovarian Cancer Risk in *BRCA1* and *BRCA2* Pathogenic Variant Carriers. Front. Oncol..

[31] Pujol P., Lyonnet D.S., Frebourg T., Blin J., Picot M.C., Lasset C., Dugast C., Berthet P., de Paillerets B.B., Sobol H. (2013). Lack of referral for genetic counseling and testing in *BRCA1/2* and Lynch syndromes: A nationwide study based on 240,134 consultations and 134,652 genetic tests. Breast Cancer Res. Treat..

[32] Thompson D., Easton D., Breast Cancer Linkage Consortium (2001). Variation in cancer risks, by mutation position, in *BRCA2* mutation carriers. Am. J. Hum. Genet..

[33] Yoshida R. (2020). Hereditary Breast and Ovarian Cancer (HBOC): Review of Its Molecular Characteristics, Screening, Treatment, and Prognosis. Breast Cancer.

[34] Gonzalez-Angulo A.M., Timms K.M., Liu S., Chen H., Litton J.K., Potter J., Lanchbury J.S., Stemke-Hale K., Hennessy B.T., Arun B.K. (2011). Incidence and outcome of *BRCA* mutations in unselected patients with triple receptor-negative breast cancer. Clin. Cancer Res..

[35] Hubalek M., Czech T., Müller H. (2017). Biological Subtypes of Triple-Negative Breast Cancer. Breast Care.

[36] Fackenthal J.D., Olopade O.I. (2007). Breast cancer risk associated with *BRCA1* and *BRCA2* in diverse populations. Nat. Rev. Cancer.

[37] Tun N.M., Villani G., Ong K., Yoe L., Bo Z.M. (2014). Risk of having *BRCA1* mutation in high-risk women with triple-negative breast cancer: A meta-analysis. Clin. Genet..

[38] Podo F., Santoro F., Di Leo G., Manoukian S., de Giacomi C., Corcione S., Cortesi L., Carbonaro L.A., Trimboli R.M., Cilotti A. (2016). Triple-Negative versus Non-Triple-Negative Breast Cancers in High-Risk Women: Phenotype Features and Survival from the HIBCRIT-1 MRI-Including Screening Study. Clin. Cancer Res..

[39] Dogan B.E., Turnbull L.W. (2012). Imaging of triple-negative breast cancer. Ann. Oncol..

[40] Foulkes W.D., Smith I.E., Reis-Filho J.S. (2010). Triple-negative breast cancer. N. Engl. J. Med..

[41] Dawson S.J., Provenzano E., Caldas C. (2009). Triple negative breast cancers: Clinical and prognostic implications. Eur. J. Cancer.

[42] Dent R., Trudeau M., Pritchard K.I., Hanna W.M., Kahn H.K., Sawka C.A., Lickley L.A., Rawlinson E., Sun P., Narod S.A. (2007). Triple-negative breast cancer: Clinical features and patterns of recurrence. Clin. Cancer Res..

[43] Noda S., Onoda N., Morisaki T., Kashiwagi S., Takashima T., Hirakawa K. (2015). The significance and the predictive factors of microscopic lymph node metastasis in patients with clinically node negative papillary thyroid cancer: A retrospective cohort study. Int. J. Surg..

[44] Phi X.-A., Houssami N., Obdeijn I.-M., Warner E., Sardanelli F., Leach M.O., Riedl C.C., Trop I., Tilanus-Linthorst M.M.A., Mandel R. (2015). Magnetic Resonance Imaging Improves Breast Screening Sensitivity in *BRCA* Mutation Carriers Age ≥ 50 Years: Evidence From an Individual Patient Data Meta-Analysis. J. Clin. Oncol..

[45] Phi X.-A., Saadatmand S., De Bock G.H., Warner E., Sardanelli F., Leach M.O., Riedl C.C., Trop I., Hooning M.J., Mandel R. (2016). Contribution of mammography to MRI screening in *BRCA* mutation carriers by *BRCA* status and age: Individual patient data meta-analysis. Br. J. Cancer.

[46] Riedl C.C., Luft N., Bernhart C., Weber M., Bernathova M., Tea M.-K.M., Rudas M., Singer C.F., Helbich T.H. (2015). Triple-modality screening trial for familial breast cancer underlines the importance of magnetic resonance imaging and questions the role of mammography and ultrasound regardless of patient mutation status, age, and breast density. J. Clin. Oncol..

[47] Metcalfe K., Eisen A., Senter L., Armel S., Bordeleau L., Meschino W.S., Pal T., Lynch H.T., Tung N.M., Kwong A. (2019). International trends in the uptake of cancer risk reduction strategies in women with a *BRCA1* or *BRCA2* mutation. Br. J. Cancer.

[48] Julian-Reynier C., Mancini J., Mouret-Fourme E., Gauthier-Villars M., Bonadona V., Berthet P., Fricker J.-P., Caron O., Luporsi E., Noguès C. (2011). Cancer risk management strategies and perceptions of unaffected women 5 years after predictive genetic testing for *BRCA1/2* mutations. Eur. J. Hum. Genet..

[49] Metcalfe K.A., Birenbaum-Carmeli D., Lubinski J., Gronwald J., Lynch H., Moller P., Ghadirian P., Foulkes W.D., Klijn J., Friedman E. (2008). International variation in rates of uptake of preventive options in *BRCA1* and *BRCA2* mutation carriers. Int. J. Cancer.

[50] de Bock G.H., Vermeulen K.M., Jansen L., Oosterwijk J.C., Siesling S., Dorrius M.D., Feenstra T., Houssami N., Greuter M.J.W. (2013). Which screening strategy should be offered to women with *BRCA1* or *BRCA2* mutations? A simulation of comparative cost-effectiveness. Br. J. Cancer.

[51] Pataky R., Armstrong L., Chia S., Coldman A.J., Kim-Sing C., McGillivray B., Scott J., Wilson C.M., Peacock S. (2013). Cost-effectiveness of MRI for breast cancer screening in *BRCA1/2* mutation carriers. BMC Cancer.

[52] Petelin L., Trainer A.H., Mitchell G., Liew D., James P.A. (2018). Cost-effectiveness and comparative effectiveness of cancer risk management strategies in *BRCA1/2* mutation carriers: A systematic review. Genet. Med..

[53] Finch A.P.M., Lubinski J., Møller P., Singer C.F., Karlan B., Senter L., Rosen B., Maehle L., Ghadirian P., Cybulski C. (2014). Impact of oophorectomy on cancer incidence and mortality in women with a *BRCA1* or *BRCA2* mutation. J. Clin. Oncol..

[54] Podfigurna-Stopa A., Czyzyk A., Grymowicz M., Smolarczyk R., Katulski K., Czajkowski K., Meczekalski B. (2016). Premature ovarian insufficiency: The context of long-term effects. J. Endocrinol. Investig..

[55] Domchek S.M., Friebel T.M., Singer C.F., Evans D.G., Lynch H.T., Isaacs C., Garber J.E., Neuhausen S.L., Matloff E., Eeles R. (2010). Association of risk-reducing surgery in *BRCA1* or *BRCA2* mutation carriers with cancer risk and mortality. JAMA.

[56] Marchetti C., De Felice F., Palaia I., Perniola G., Musella A., Musio D., Muzii L., Tombolini V., Panici P.B. (2014). Risk-reducing salpingo-oophorectomy: A meta-analysis on impact on ovarian cancer risk and all cause mortality in BRCA 1 and BRCA 2 mutation carriers. BMC Women’s Health.

[57] Rebbeck T.R., Kauff N.D., Domchek S.M. (2009). Meta-analysis of Risk Reduction Estimates Associated with Risk-Reducing Salpingo-oophorectomy in *BRCA1* or *BRCA2* Mutation Carriers. J. Natl. Cancer Inst..

[58] Singer C.F., Tea M.K., Pristauz G., Hubalek M., Rappaport C., Riedl C.C., Helbich T.H. (2015). Clinical Practice Guideline for the prevention and early detection of breast and ovarian cancer in women from HBOC (hereditary breast and ovarian cancer) families. Wien. Klin. Wochenschr..

[59] Mancini J., Mouret-Fourme E., Noguès C., Julian-Reynier C. (2015). Impact of *BRCA1/2* mutation on young women’s 5-year parenthood rates: A prospective comparative study (GENEPSO-PS cohort). Fam. Cancer.

[60] Chan J., Johnson L.N., DiGiovanni L., Voong C., Sammel M.D., Domchek S.M., Gracia C. (2015). Reproductive decision-making in patients diagnosed with BRCA mutations. Fertil. Steril..

[61] Peccatori F.A., Mangili G., Bergamini A., Filippi F., Martinelli F., Ferrari F., Noli S., Rabaiotti E., Candiani M., Somigliana E. (2018). Fertility preservation in women harboring deleterious BRCA mutations: Ready for prime time?. Hum. Reprod..

[62] Kim J., Gammon M.D., Skrzynia C., Mersereau J.E. (2013). BRCA mutation carriers: A new target population for fertility preservation consultation and treatment. Fertil. Steril..

[63] Gunnala V., Fields J., Irani M., D’Angelo D., Xu K., Schattman G., Rosenwaks Z. (2019). BRCA carriers have similar reproductive potential at baseline to noncarriers: Comparisons in cancer and cancer-free cohorts undergoing fertility preservation. Fertil. Steril..

[64] Grynberg M., Raad J., Comtet M., Vinolas C., Cédrin-Durnerin I., Sonigo C. (2018). Fertility preservation in *BRCA*-mutated women: When and how?. Future Oncol..

[65] Milne R.L., Antoniou A.C. (2016). Modifiers of breast and ovarian cancer risks for *BRCA1* and *BRCA2* mutation carriers. Endocr. Relat. Cancer.

[66] Osorio A., Milne R.L., Pita G., Peterlongo P., Heikkinen T., Simard J., Chenevix-Trench G., Spurdle A.B., Beesley J., Chen X. (2009). Evaluation of a candidate breast cancer associated SNP in ERCC4 as a risk modifier in *BRCA1* and *BRCA2* mutation carriers. Results from the Consortium of Investigators of Modifiers of *BRCA1*/*BRCA2* (CIMBA). Br. J. Cancer.

[67] Mulligan A.M., Couch F.J., Barrowdale D., Domchek S.M., Eccles D., Nevanlinna H., Ramus S.J., Robson M., Sherman M., Spurdle A.B. (2011). Common breast cancer susceptibility alleles are associated with tumour subtypes in *BRCA1* and *BRCA2* mutation carriers: Results from the Consortium of Investigators of Modifiers of *BRCA1/2*. Breast Cancer Res..

[68] Antoniou A.C., Kartsonaki C., Sinilnikova O.M., Soucy P., McGuffog L., Healey S., Lee A., Peterlongo P., Manoukian S., Peissel B. (2011). Common alleles at 6q25.1 and 1p11.2 are associated with breast cancer risk for *BRCA1* and *BRCA2* mutation carriers. Hum. Mol. Genet..

[69] Bugrein H. (2015). Genotype and phenotype correlation of breast cancer in BRCA carriers and non-carriers. J. Cancer Sci. Ther..

[70] Bujassoum S.M., Bugrein H.A., Al Sulaiman R. (2017). Genotype and Phenotype Correlation of Breast Cancer in BRCA Mutation Carriers and Non-Carriers. J. Cancer Sci. Ther..

[71] Lecarpentier J., Noguès C., Mouret-Fourme E., Buecher B., Gauthier-Villars M., Stoppa-Lyonnet D., Bonadona V., Fricker J.-P., Berthet P., Caron O. (2015). Breast Cancer Risk Associated with Estrogen Exposure and Truncating Mutation Location in *BRCA1/2* Carriers. Cancer Epidemiol. Biomark. Prev..

[72] Andrieu N., Easton D.F., Chang-Claude J., Rookus M.A., Brohet R., Cardis E., Antoniou A.C., Wagner T., Simard J., Evans G. (2006). Effect of chest X-rays on the risk of breast cancer among *BRCA1/2* mutation carriers in the international *BRCA1/2* carrier cohort study: A report from the EMBRACE, GENEPSO, GEO-HEBON, and IBCCS Collaborators’ Group. J. Clin. Oncol..

[73] Narod S.A. (2006). Modifiers of risk of hereditary breast cancer. Oncogene.

[74] Friebel T.M., Domchek S.M., Rebbeck T.R. (2014). Modifiers of cancer risk in *BRCA1* and *BRCA2* mutation carriers: Systematic review and meta-analysis. J. Natl. Cancer Inst..

[75] Mavaddat N., Michailidou K., Dennis J., Lush M., Fachal L., Lee A., Tyrer J.P., Chen T.-H., Wang Q., Bolla M.K. (2019). Polygenic Risk Scores for Prediction of Breast Cancer and Breast Cancer Subtypes. Am. J. Hum. Genet..

[76] Barnes D.R., Rookus M.A., McGuffog L., Leslie G., Mooij T.M., Dennis J., Mavaddat N., Adlard J., Ahmed M., Aittomäki K. (2020). Polygenic risk scores and breast and epithelial ovarian cancer risks for carriers of *BRCA1* and *BRCA2* pathogenic variants. Genet. Med..

[77] Gallagher S., Hughes E., Wagner S., Tshiaba P., Rosenthal E., Roa B.B., Kurian A.W., Domchek S.M., Garber J., Lancaster J. (2020). Association of a Polygenic Risk Score with Breast Cancer Among Women Carriers of High- and Moderate-Risk Breast Cancer Genes. JAMA Netw. Open.

[78] Mars N., Koskela J.T., Ripatti P., Kiiskinen T.T.J., Havulinna A.S., Lindbohm J.V., Ahola-Olli A., Kurki M., Karjalainen J., Palta P. (2020). Polygenic and clinical risk scores and their impact on age at onset and prediction of cardiometabolic diseases and common cancers. Nat. Med..

[79] Levy D.E., Garber J.E., Shields A.E. (2009). Guidelines for genetic risk assessment of hereditary breast and ovarian cancer: Early disagreements and low utilization. J. Gen. Intern. Med..

[80] George A., Kaye S., Banerjee S. (2017). Delivering widespread *BRCA* testing and PARP inhibition to patients with ovarian cancer. Nat. Rev. Clin. Oncol..

[81] U.S. Breast Cancer Statistics. https://www.breastcancer.org/symptoms/understand_bc/statistics.

[82] Institut National Du Cancer Epidémiologie des Cancers—Les chiffres du Cancer en France. https://www.e-cancer.fr/Professionnels-de-sante/Les-chiffres-du-cancer-en-France/Epidemiologie-des-cancers.

[83] Pearce N. (2012). Classification of epidemiological study designs. Int. J. Epidemiol..

[84] Kass P.H. (2014). Modern Epidemiological Study Designs. Handbook of Epidemiology.

