# Supplementary material for: No Association of Early-Onset Breast or Ovarian Cancer with Early-Onset Cancer in Relatives in BRCA1 or BRCA2 Mutation Families
Source: Genes (Basel). 2021 Jul 20;12(7):1100. doi: 10.3390/genes12071100 (PMC8305427; doi:10.3390/genes12071100)
Supplement: Supplementary file 1 [file genes-12-01100-s001.zip › genes-1249604-supplementary/Table S1. Patients and Families Characteristics.pptx]

## Slide 1
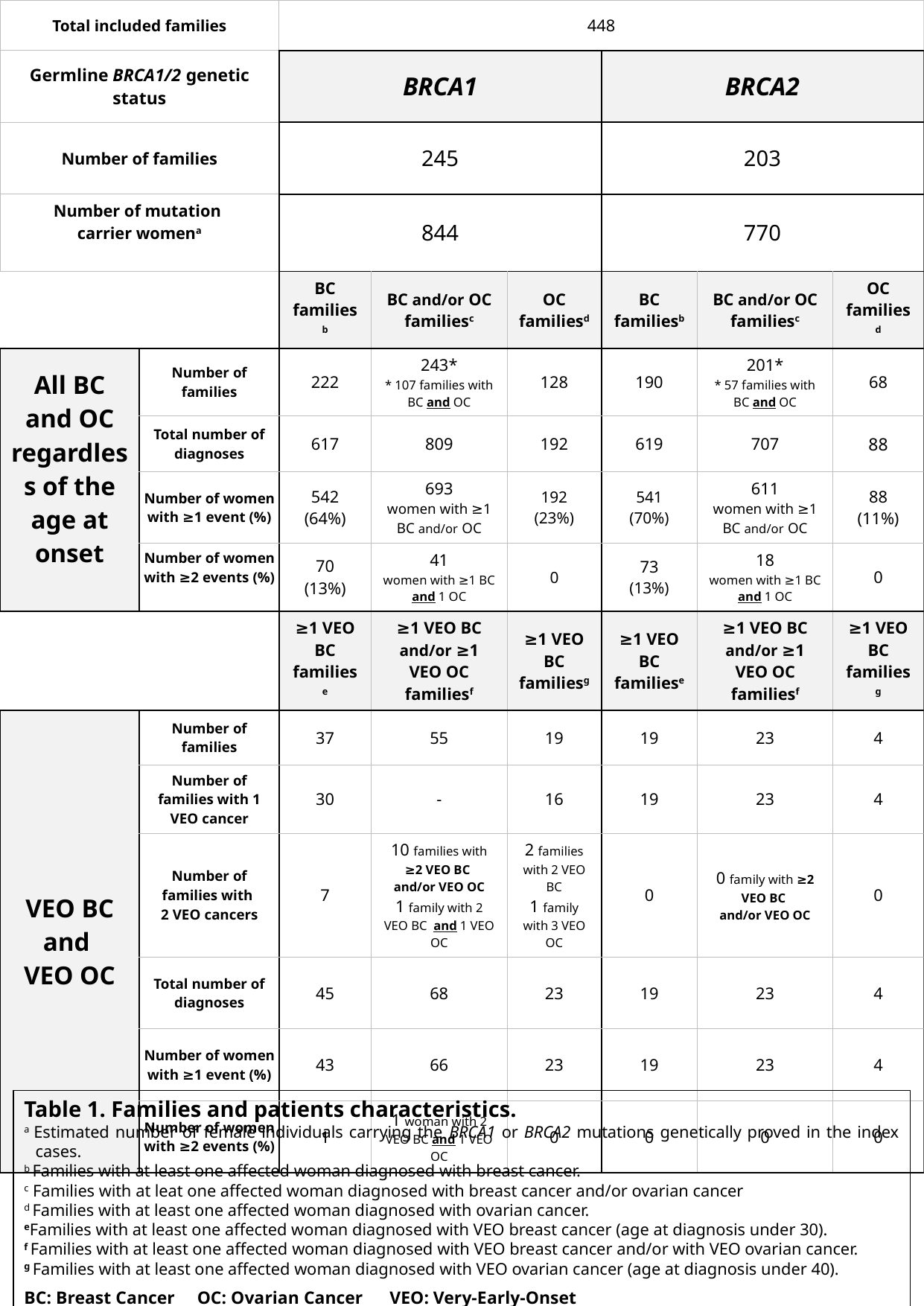

| Total included families | | 448 | | | | | |
| --- | --- | --- | --- | --- | --- | --- | --- |
| Germline BRCA1/2 genetic status | | BRCA1 | | | BRCA2 | | |
| Number of families | | 245 | | | 203 | | |
| Number of mutation carrier womena | | 844 | | | 770 | | |
| | | BC familiesb | BC and/or OC familiesc | OC familiesd | BC familiesb | BC and/or OC familiesc | OC familiesd |
| All BC and OC regardless of the age at onset | Number of families | 222 | 243\* \* 107 families with BC and OC | 128 | 190 | 201\* \* 57 families with BC and OC | 68 |
| | Total number of diagnoses | 617 | 809 | 192 | 619 | 707 | 88 |
| | Number of women with ≥1 event (%) | 542 (64%) | 693 women with ≥1 BC and/or OC | 192 (23%) | 541 (70%) | 611 women with ≥1 BC and/or OC | 88 (11%) |
| | Number of women with ≥2 events (%) | 70 (13%) | 41 women with ≥1 BC and 1 OC | 0 | 73 (13%) | 18 women with ≥1 BC and 1 OC | 0 |
| | | ≥1 VEO BC familiese | ≥1 VEO BC and/or ≥1 VEO OC familiesf | ≥1 VEO BC familiesg | ≥1 VEO BC familiese | ≥1 VEO BC and/or ≥1 VEO OC familiesf | ≥1 VEO BC familiesg |
| VEO BC and VEO OC | Number of families | 37 | 55 | 19 | 19 | 23 | 4 |
| | Number of families with 1 VEO cancer | 30 | - | 16 | 19 | 23 | 4 |
| | Number of families with 2 VEO cancers | 7 | 10 families with ≥2 VEO BC and/or VEO OC 1 family with 2 VEO BC and 1 VEO OC | 2 families with 2 VEO BC 1 family with 3 VEO OC | 0 | 0 family with ≥2 VEO BC and/or VEO OC | 0 |
| | Total number of diagnoses | 45 | 68 | 23 | 19 | 23 | 4 |
| | Number of women with ≥1 event (%) | 43 | 66 | 23 | 19 | 23 | 4 |
| | Number of women with ≥2 events (%) | 1 | 1 woman with 2 VEO BC and 1 VEO OC | 0 | 0 | 0 | 0 |
Table 1. Families and patients characteristics.
a Estimated number of female individuals carrying the BRCA1 or BRCA2 mutations genetically proved in the index cases.
b Families with at least one affected woman diagnosed with breast cancer.
c Families with at leat one affected woman diagnosed with breast cancer and/or ovarian cancer
d Families with at least one affected woman diagnosed with ovarian cancer.
eFamilies with at least one affected woman diagnosed with VEO breast cancer (age at diagnosis under 30).
f Families with at least one affected woman diagnosed with VEO breast cancer and/or with VEO ovarian cancer.
g Families with at least one affected woman diagnosed with VEO ovarian cancer (age at diagnosis under 40).
BC: Breast Cancer OC: Ovarian Cancer VEO: Very-Early-Onset
