# Supplementary material for: No Association of Early-Onset Breast or Ovarian Cancer with Early-Onset Cancer in Relatives in BRCA1 or BRCA2 Mutation Families
Source: Genes (Basel). 2021 Jul 20;12(7):1100. doi: 10.3390/genes12071100 (PMC8305427; doi:10.3390/genes12071100)
Supplement: Supplementary file 1 [file genes-12-01100-s001.zip › genes-1249604-supplementary/Table S2. Median age of BC diagnosis in relatives of women with VEO BC.pdf]

Table S2.

Median age of Breast Cancer diagnosis in relatives of women with VEO BC

➢ BRCA1

	VEO BC within family YES / NO					
**Variable**	**Parameters**	**Total population**	**Subset 0:NO**	**Subset 1:YES**	**Test**	**p**
Average within family of age of breast cancer diagnosis of relatives	Average (± SD)	47.32 (±9.07)	47.18 (±8.514)	48.22 (±12.14)	WMW	0.84
	Median (Q25;Q75)	47.00 (40.00;54.00)	46.75 (40.75 ; 54.00)	47.00 (39.00 ; 53.63)		
	[Min;Max]	[31.00;80.00]	[31.00 ; 75.00]	[32.00 ; 80.00]		
	N	204	176	28		

➢ BRCA2

	VEO BC within family YES / NO					
**Variable**	**Parameters**	**Total population**	**Subset 0:NO**	**Subset 1:YES**	**Test**	**p**
Average within family of age of breast cancer diagnosis of relatives	Average (± SD)	48.93 (±8.82)	48.71 (±8.55)	50.77 (±10.96)	WMW	0.39
	Median (Q25;Q75)	48.20 (42.67;54.00)	47.71 (42.71 ; 54.17)	51.50 (42.00 ; 54.00)		
	[Min;Max]	[31.00;82.00]	[33.00 ; 77.00]	[31.00 ; 82.00]		
	N	183	164	19		

Median age of Ovarian Cancer diagnosis in relatives of women with VEO OC

➢ BRCA1

	VEO OC within family YES / NO					
**Variable**	**Parameters**	**Total population**	**Subset 0:NO**	**Subset 1:YES**	**Test**	**p**
Average within family of age of ovarian cancer diagnosis of relatives	Average (± SD)	55.87 (±9.31)	55.88 (±8.92)	55.81 (±12.84)	WMW	0.81
	Median (Q25;Q75)	55.25 (49.00;63.00)	55.50 (49.00 ; 62.00)	54.00 (46.00 ; 69.00)		
	[Min;Max]	[34.00;90.00]	[41.00 ; 90.00]	[34.00 ; 73.00]		
	N	110	99	11		

➢ BRCA2

	VEO OC within family YES / NO					
**Variable**	**Parameters**	**Total population**	**Subset 0:NO**	**Subset 1:YES**	**Test**	**p**
Average within family of age of ovarian cancer diagnosis of relatives	Average (± SD)	62.29 (±10.00)	62.41 (±10.06)	56.00 (±.)	.	.
	Median (Q25;Q75)	60.50 (54.50;70.00)	61.00 (54.50 ; 70.00)	56.00 (56.00 ; 56.00)		
	[Min;Max]	[43.00;83.00]	[43.00 ; 83.00]	[56.00 ; 56.00]		
	N	54	53	1		

Mixed-effects model

Age at breast cancer diagnosis in relatives

- BRCA1

Solution for fixed effects						
Effect	VEOBC within family YN	Estimation	Erreur type	DDL	Valeur du test t	Pr > |t|
Intercept		47.5770	1.4894	202	31.94	<.0001
family_VEOBC_YN_b	0:NO	-0.1481	1.6199	215	-0.09	0.9272
family_VEOBC_YN_b	1:YES	0	.	.	.	.

Least-Squares Means							
Effect	VEOBC within family YN	Estimation	Erreur type	SD calc	DDL	Valeur du test t	Pr > |t|
family_VEOBC_YN_b	0:NO	47.4288	0.6369	8,449	215	74.46	<.0001
family_VEOBC_YN_b	1:YES	47.5770	1.4894	7,881	215	31.94	<.0001

- BRCA2

Solution for fixed effects						
Effect	VEOBC within family YN	Estimation	Erreur type	DDL	Valeur du test t	Pr > |t|
Intercept		53.1654	1.6054	181	33.12	<.0001
family_VEOBC_YN_b	0:NO	-3.9086	1.7091	249	-2.29	0.0230
family_VEOBC_YN_b	1:YES	0	.	.	.	.

Least-Squares Means							
Effect	VEOBC within family YN	Estimation	Erreur type	SD calc	DDL	Valeur du test t	Pr > |t|
family_VEOBC_YN_b	0:NO	49.2568	0.5864	7,509	249	84.00	<.0001
family_VEOBC_YN_b	1:YES	53.1654	1.6054	6,997	249	33.12	<.0001

Age at ovarian cancer diagnosis in relatives

- BRCA1

Solution for fixed effects						
Effect	VEOOC within family YN	Estimation	Erreur type	DDL	Valeur du test t	Pr > |t|
Intercept		53.2289	2.6174	108	20.34	<.0001
family_VEOOC_YN_b	0:NO	2.5817	2.7768	36	0.93	0.3587
family_VEOOC_YN_b	1:YES	0	.	.	.	.

Least-Squares Means							
**Effect**	**VEOOC within family YN**	**Estimation**	**Erreur type**	**SD calc**	**DDL**	**Valeur du test t**	**Pr > |t|**
**family_VEOOC_YN_b**	0:NO	55.8106	0.9274	9,227	36	60.18	<.0001
**family_VEOOC_YN_b**	1:YES	53.2289	2.6174	8,681	36	20.34	<.0001
