# Supplementary material for: No Association of Early-Onset Breast or Ovarian Cancer with Early-Onset Cancer in Relatives in BRCA1 or BRCA2 Mutation Families
Source: Genes (Basel). 2021 Jul 20;12(7):1100. doi: 10.3390/genes12071100 (PMC8305427; doi:10.3390/genes12071100)
Supplement: Supplementary file 1 [file genes-12-01100-s001.zip › genes-1249604-supplementary/Table S3. Distributions age at diagnosis of BC and OC of relatives.pdf]

Table S3. Distribution of ages at diagnosis of breast cancer and ovarian cancer of relatives of women with a VEO cancer compared with relatives of women without VEO cancer.

Distribution of ages at diagnosis of breast cancer of relatives of women with a VEO BC compared with relatives of women without VEO BC.

- BRCA1

			VEO BC within family YES/NO			
		Total population	0:NO	1:YES		
variable	modalities	n (%col)	n (%col)	n (%col)	Test	p
Age at BREAST CANCER DIAGNOSIS of affected relatives (5 age classes)	1:31-35years	44 (10.68)	40 (11.30)	4 (6.90)	CHI2	0.07
	2:36-40years	98 (23.79)	83 (23.45)	15 (25.86)		
	3:41-45years	70 (16.99)	66 (18.64)	4 (6.90)		
	4:46-50years	68 (16.50)	53 (14.97)	15 (25.86)		
	5:>50years	132 (32.04)	112 (31.64)	20 (34.48)		
	N	412	354 (100.00)	58 (100.00)		

			VEO BC within family YES/NO			
		Total population	0:NO	1:YES		
variable	modalities	n (%col)	n (%col)	n (%col)	Test	p
Age at BREAST CANCER DIAGNOSIS of affected relatives (4 age classes)	1:31-35years	44 (10.68)	40 (11.30)	4 (6.90)	CHI2	0.76
	2:36-40years	98 (23.79)	83 (23.45)	15 (25.86)		
	3:41-50years	138 (33.50)	119 (33.62)	19 (32.76)		
	4:>50years	132 (32.04)	112 (31.64)	20 (34.48)		
w	N	412	354 (100.00)	58 (100.00)		

- BRCA2

			VEO BC within family YES/NO			
		Total population	0:NO	1:YES		
**variable**	**modalities**	**n (%col)**	**n (%col)**	**n (%col)**	**Test**	**p**
Age at BREAST CANCER DIAGNOSIS of affected relatives (5 age classes)	1:31-35years	44 (10.19)	40 (10.50)	4 (7.84)	CHI2	0.88
	2:36-40years	65 (15.05)	58 (15.22)	7 (13.73)		
	3:41-45years	62 (14.35)	56 (14.70)	6 (11.76)		
	4:46-50years	84 (19.44)	72 (18.90)	12 (23.53)		
	5:>50years	177 (40.97)	155 (40.68)	22 (43.14)		
	N	432	381 (100.00)	51 (100.00)		

			VEO BC within family YES/NO			
		Total population	0:NO	1:YES		
**variable**	**modalities**	**n (%col)**	**n (%col)**	**n (%col)**	**Test**	**p**
Age at BREAST CANCER DIAGNOSIS of affected relatives (4 age classes)	1:31-35years	44 (10.19)	40 (10.50)	4 (7.84)	CHI2	0.92
	2:36-40years	65 (15.05)	58 (15.22)	7 (13.73)		
	3:41-50years	146 (33.80)	128 (33.60)	18 (35.29)		
	4:>50years	177 (40.97)	155 (40.68)	22 (43.14)		
	N	432	381 (100.00)	51 (100.00)		

Distribution of ages at diagnosis of ovarian cancer of relatives of women with a VEO OC compared with relatives of women without VEO OC.

- BRCA1

			VEO OC within family YES/NO			
		Total population	0:NO	1:YES		
variable	modalities	n (%col)	n (%col)	n (%col)	Test	p
Age at OVARIAN CANCER DIAGNOSIS of affected relatives (5 age classes)	1:41-45years	22 (15.49)	20 (15.63)	2 (14.29)	FISHER	0.77
	2:46-50years	31 (21.83)	27 (21.09)	4 (28.57)		
	3:51-55years	20 (14.08)	19 (14.84)	1 (7.14)		
	4:56-60years	23 (16.20)	22 (17.19)	1 (7.14)		
	5:>60years :	46 (32.39)	40 (31.25)	6 (42.86)		
	N	142	128 (100.00)	14 (100.00)		

			VEO OC within family YES/NO			
		Total population	0:NO	1:YES		
variable	modalities	n (%col)	n (%col)	n (%col)	Test	p
Age at OVARIAN CANCER DIAGNOSIS of affected relatives (4 age classes)	1:41-45years	22 (15.49)	20 (15.63)	2 (14.29)	FISHER	0.51
	2:46-50years	31 (21.83)	27 (21.09)	4 (28.57)		
	3:51-60years	43 (30.28)	41 (32.03)	2 (14.29)		
	4:>60years	46 (32.39)	40 (31.25)	6 (42.86)		
	N	142	128 (100.00)	14 (100.00)		
