# Supplementary material for: No Association of Early-Onset Breast or Ovarian Cancer with Early-Onset Cancer in Relatives in BRCA1 or BRCA2 Mutation Families
Source: Genes (Basel). 2021 Jul 20;12(7):1100. doi: 10.3390/genes12071100 (PMC8305427; doi:10.3390/genes12071100)
Supplement: Supplementary file 1 [file genes-12-01100-s001.zip › genes-1249604-supplementary/Table S5. Cochran-Mantel-Haenszel.pdf]

Table S5. Cochran-Mantel-Haenszel statistics

Comparison of early onset BC (31-35) within family and number of VEO BC occurrence

- BRCA1

Statistique de Cochran-Mantel-Haenszel (Basé sur les scores de tables)				
**Statistique**	**Hypothèse alternative**	**DDL**	**Valeur**	**Prob**
**1**	**Corrélation non nulle**	1	0.0255	0.8731

- ➔ Number of early onset BC in borderline age class (31-35) within family
- ➔ Number of VEO BC within family
- ➔ Weithing based on number of BC within family

Not significant correlation

- BRCA2

Statistique de Cochran-Mantel-Haenszel (Basé sur les scores de tables)				
**Statistique**	**Hypothèse alternative**	**DDL**	**Valeur**	**Prob**
**1**	**Corrélation non nulle**	1	0.3238	0.5693

- ➔ Number of early onset BC in borderline age class (31-35) within family
- ➔ Number of VEO BC within family
- ➔ Weithing based on number of BC within family

Not significant correlation

Comparison of early onset OC (41-45) within family and number of VEO OC occurrence

➢ BRCA1

Statistique de Cochran-Mantel-Haenszel (Basé sur les scores de tables)				
**Statistique**	**Hypothèse alternative**	**DDL**	**Valeur**	**Prob**
**1**	**Corrélation non nulle**	1	0.5749	0.4483

- ➔ Number of early onset OC in borderline age class (41-45) within family
- ➔ Number of VEO OC within family
- ➔ Weithing based on number of 0C within family

Not significant correlation
